# RNA sequencing to characterize transcriptional changes of sexual maturation and mating in the female oriental fruit fly *Bactrocera dorsalis*

**DOI:** 10.1186/s12864-016-2532-6

**Published:** 2016-03-05

**Authors:** Weiwei Zheng, Deye Luo, Fangyu Wu, Jialu Wang, Hongyu Zhang

**Affiliations:** State Key Laboratory of Agricultural Microbiology, Hubei Key Laboratory of Insect Resource Application and Sustainable Pest Control and Institute of Urban and Horticultural Pests, College of Plant Science and Technology, Huazhong Agricultural University, Wuhan, 430070 Hubei People’s Republic of China

**Keywords:** RNAseq, Transcriptome, Tephritid, Maturity, Post-mating, Reproduction, Gene expression

## Abstract

**Background:**

Female reproductive potential plays a significant role in the survival and stability of species, and sexual maturation and mating processes are crucial. However, our knowledge of the reproductive genes involved in sexual maturation and mating has been largely limited to model organisms. The oriental fruit fly *Bactrocera dorsalis* is a highly invasive agricultural pest, known to cause major economic losses; thus, it is of great value to understand the transcriptional changes involved in sexual maturation and mating processes as well as the related genes. Here, we used a high-throughput sequencing method to identify multiple genes potentially involved in sexual maturation and mating in female *B. dorsalis*.

**Results:**

We sequenced 39,999 unique genes with an average length of 883 bp. In total, 3264 differentially expressed genes (DEGs) were detected between mature virgin and immature *Bactrocera dorsalis* libraries, whereas only 83 DEGs were identified between flies that had mated or were mature virgins. These DEGs were functionally annotated using the GO and KEGG pathway annotation tools. Results showed that the main GO terms associated with the DEGs from the mature virgin vs. immature groups were primarily assigned to the metabolic and developmental processes, which we focused on, whereas those from the mated vs. mature virgin group largely belonged to the response to stimulus and immune system processes. Additionally, we identified multiple DEGs during sexual maturation that are involved in reproduction, and expression pattern analysis revealed that the majority DEGs detected were highly enriched in those linked to the ovaries or fat bodies. Several mating responsive genes differentially expressed after mating were also identified, and all antimicrobial peptides detected were highly enriched in fat body and significantly up-regulated approximately 2- to 10-fold at 24 h after mating.

**Conclusion:**

This study supplied female reproductive genes involved in sexual maturation and the post-mating response in *B. dorsalis,* based on RNA-seq. Our data will facilitate molecular research related to reproduction and provide abundant target genes for effective control of this agricultural pest.

**Electronic supplementary material:**

The online version of this article (doi:10.1186/s12864-016-2532-6) contains supplementary material, which is available to authorized users.

## Background

Tephritid fruit flies, especially those belonging to the genus *Bactrocera*, are globally important due to their destructive impact on agriculture. Their high reproductive ability is an important factor that leads to enhanced survival rates and potential to multiply. Reproductive biology has been well studied in tephritid species, including *Bactrocera tryoni* (Froggatt), *Bactrocera cucurbitae* (Coquillett) and *Ceratitis capitata* (Wiedemann), but most reports have mainly focused on the processes at the physiological and behavioral levels [1–3]. *Bactrocera dorsalis* (Hendel) (the oriental fruit fly), which is one among the important *Bactrocera* species, is a highly invasive agricultural pest that is currently distributed across most Asian countries and in a number of Pacific Islands. Adults can lay eggs on various types of host plants, where the hatched larvae subsequently feed and cause crop loss [4]. Some factors that regulate the fecundity and mating behavior of *B. dorsalis* females have been previously studied, such as methyl eugenol-fed males and multiple mates, etc. [5]. Although the *B. dorsalis* transcriptome from different developmental stages of the whole body as well as specific tissue in males is available on NCBI, large-scale molecular analysis of reproduction in *B. dorsalis* females remains limited [6–10]. As an invasive agricultural pest with a wide host range and high fecundity, it is essential to investigate the reproductive biology of *B. dorsalis* females at the molecular level in order to formulate simple and effective strategies for agricultural pest control.

Recently, transcriptional changes in several genes that may be involved in female sexual maturity and mating were identified in *C. capitata* based on microarray data [11]; however, the cDNA library of *C. capitata* is limited to the head of female adult. Additionally, it is worth noting that the reproductive strategy of *B. dorsalis* is different from that of *C. capitata*. For instance, medfly reach sexual maturity rapidly within 2–3 days after emergence, whereas it takes approximately 2 weeks for the oriental fruit fly to complete the transformation after emergence to maturity [12, 13]. Therefore, our study is planned because both species have different mating strategy and that the methodology followed in previous study provided only limited resources.

Female reproductive potential significantly contributed to the multiply and stability of species, which mainly depends on the process of sexual maturation and mating [14]. In most holometabolous insects, adults emerge from the pupal stage as sexually immature individuals. After they ingest a food source, they become sexually mature within several days dependent on the species [14]. During the development of the oocyte, the ovary becomes mature and then when females reach sexual maturity they began to mate and oviposit [15]. Until now, studies on sexual maturity have mainly concentrated on the model species whose genomes have been sequenced except *C. capitata*, including *Drosophila melanogaster*, *Apis mellifera*, *Aedes aegypti* and *Blattella germanica* [16–20]. However, these reports on model species mainly focused on the protein vitellogenin and information on other proteins that play roles in reproduction is lacking. While genes involved in female adult reproductive maturation, including *EcR*, *USP* and *InR*, have been identified in *Tribolium castaneum* using microarrays, they are mainly the early genes in the signal transduction pathway, and little is known about the other genes, especially the late or effect genes [21].

Successful mating behavior and the following egg-laying is essential for sexual reproduction in insect, especially for its multiply. Female reproductive process has been revealed to be greatly affected by male reproductive gland secretion during copulation. Reports in *C. capitata* and *Bactrocera tryoni* showed the effect of mating on remating propensity, refractory period and fecundity of females [22–24]. In *D. melanogaster*, it was reported that mating regulated ovulation and spawning as well as other processes in mated females, such as, inducing innate immunity, acceleration of reproductive maturation and egg production [25–28]. The sex peptide receptor, which mediates the post-mating switch in females, has been reported in *D. melanogaster*, *Helicoverpa armigera and B. dorsalis* [4, 29–31]. However, the other factors involved in this process are still largely unknown. Genome-wide research into the post-mating response in females has been performed in model species such as *D. melanogaster*, the honeybee queen *Apis mellifera*, and *C. capitata*, which have revealed that the post-mating response differs among species as well as the time following mating [11, 25, 28, 32–34]. Regardless, there is no such information available for *B. dorsalis*, and the transcriptional changes and genes involved in this process is also still unknown.

In the present study, the transcriptome of the whole body of the female *B. dorsalis* were sequenced at three different physiological stages (immature, mature virgins and mated individuals) using the Illumina HiSeq 2500 system, and assembled based on our previously established transcriptome of *B. dorsalis* [6]. Furthermore, analysis of the DEGs between mature virgins and immature individuals was performed in order to identify the transcripts involved in the process of sexual maturation. An additional comparison between mated individuals and mature virgins was also carried out to identify the genes that respond to mating. Tissue specific expression profiles of the differentially expressed genes from these two comparisons were analyzed by qRT-PCR. Finally, the expression levels of the genes involved in the post-mating response at different time points were also obtained.

## Methods

### Insect culture

*B. dorsalis* were cultured in our lab under a 12 h light/dark cycle at 27 °C. Adult flies were reared on artificial diets composed of 25 % yeast extract and 75 % sugar [35].

### Sample preparation

Newly emerged adults were collected and sexed within 12 h after emergence, and both sexes were subsequently maintained in separate cages. As male and female oriental fruit flies require 14 days to reach sexual maturity under standard rearing conditions, 1- to 2-day-old (1–2 days) individuals were considered immature while 14-day-old (14 days) individuals were considered sexually mature. Twenty-four hours after emergence, the immature female flies were chilled briefly and RNA was extracted from the whole bodies of 10 individuals using TRIzol regent (Invitrogen, Carlsbad, CA, USA). Similarly, mature virgin samples were collected 14 days after emergence and total RNA was isolated from the whole bodies of 10 mature virgin females.

To obtain mated flies, approximately 100 fourteen-day-old virgin flies of each sex were placed into a 30 cm^3^ cage shortly before dark as *B. dorsalis* mating occurs during dusk [36]. When copulation was observed, mating pairs were isolated and placed in small vials about 14 cm in width and 23 cm in height with 11.5 cm diameter. Notably, only pairs that mated for at least 90 min were used in further experiments in order to avoid false mating with little or no sperm transfer. Copulation terminated naturally and the mated pairs were placed in separate cages according to sex. RNA was extracted from the whole bodies of 10 mated females the following day (24 h after mating) with the same procedure as was used for the virgin flies. RNA samples were treated to remove any contaminating DNA with the DNA-free kit (Ambion, Foster City, CA, USA).

### Sequencing

Total RNA from each sample was isolated using TRIzol reagent (Invitrogen) following the manufacturer’s instructions. The concentration and integrity of total RNA were determined using the 2100 Bioanalyzer (Agilent, Santa Clara, CA, USA). Total RNA was then incubated with 10 U DNase I (Takara, Shiga, Japan) at 37 °C for 30 min, and purified using the Dynabeads® Oligo (dT)25 (Thermo Fisher, Waltham, MA, USA) following the manufacturer’s instructions. To ensure the accuracy of the results, we conducted two independent replicates for each sample, specifically immature (1, 2), mature virgin (1, 2) and mated (1, 2) individuals. For each case, the following validation described below was performed.

For cDNA library construction 100 ng purified mRNA was used using NEBNext® UltraTM RNA Library Prep Kit for Illumina sequencing (New England Biolabs, Ipswich, MA, USA). First-strand cDNA was synthesized using ProtoScript II Reverse Transcriptase. After incubation, Second Strand Synthesis Enzyme Mix was added for the synthesis of the second strand of the cDNA. Double-stranded cDNA was then purified using AMPure XP Beads (Beckman Coulter, Brea, CA, USA), followed by an end repair step using End Prep Enzyme Mix. After adaptor ligation and further purification, the cDNA library was obtained. To verify the quality of the library, it was checked using three different methods, including Qubit (Thermo Fisher) quantitative analysis, 2 % agarose gel electrophoresis, and high-sensitivity DNA chip determination.

The cDNA libraries were then used for cluster generation with the TruSeq PE Cluster Kit (Illumina, San Diego, CA, USA) and sequenced on an Illumina HiSeq^™^ 2500 instrument with paired-end sequencing.

### Sequence assembly and annotation

Raw data were first filtered by removing adaptor sequences and low quality reads using FASTX-Toolkit (http://hannonlab.cshl.edu/fastx_toolkit/), and then assembled into contigs using Trinity (v2013-02-25) with N bases as the default assembly parameters. Finally, redundant sequences were removed while those remaining constituted the transcriptome used in downstream analyses. Furthermore, depth and coverage of sequencing was evaluated. The assembly data have been submitted to NCBI as a TSA under the accession number GEEA00000000.

All contigs were annotated with GetORF of the EMBOSS package [37]. The ORF of each predicted protein was used in a BLASTp search against the Swiss-Prot and the NCBI nr databases, with the e-value threshold set to 10^−5^. Gene Ontology (GO) annotations were performed with GoPipe [38]. Predicted proteins were first used for a BLASTp search against the Swiss-Prot and TrEMBL databases with an E-value cut-off of 10^−5^, and then the results were analyzed by GoPipe based with the gene2go software. The KOG (Eukaryotic Orthologous Groups) and KEGG pathway annotations were also analyzed using the Cluster of Orthologous Groups database and the Kyoto Encyclopedia of Genes and Genomes database, respectively [39].

### Differential expression analysis

For the identification of genes that were possibly involved in reproduction, those that were differentially expressed at the various physiological stages of adults were analyzed. Two comparisons of the expression level of paired sets of the three samples were performed between mature virgin vs. immature and mated vs. mature virgin individuals. The number of reads for contigs from each sample was first converted to Reads per Kilobase per Million (RPKM), and then the MARS model (MA-plot-based method with Random Sampling model) in the DEGseq package was used to calculate the expression abundance of each contig [40]. A false discovery rate (FDR) < 0.001 was considered to indicate significant expression abundance. Additionally, differentially expressed genes were further used for GO and KEGG analysis to identify the pathways that the DEGs were predicted to be involved in. Relativity analysis was performed to evaluate the stability and reliability of the replicates. GOEAST was used for GO term enrichment analysis [41]. The relative significant level of enrichment of the differently expressed gene to all genes was calculated with hypergeometric statistical test method, then the GO term whose FDR < 0.001 was considered to be significant. For pathway enrichment analysis, a similar method was used. The KEGG pathway was considered as a unit, and its relative significant level of enrichment of the differently expressed pathway to whole genome was calculated.

### Quantitative real-time PCR (qRT-PCR) verification

Selected DEG data was validated by qRT-PCR using the SYBR Premix Ex Taq kit (Takara) according to the manufacturer’s instructions with a real-time thermal cycler (Bio-Rad, Hercules, CA). TRIzol reagent (Invitrogen) was used to extract total RNA from *B. dorsalis* at three different physiological stages, including the immature (newly emergent within 24 h), mature virgin (14-day-old before mating), and mated (14-day-old post-mating). At least 10 insects were collected for each sample. The first strand cDNA was obtained from 2 μg of total RNA using M-MLV Reverse Transcriptase (Takara) with the primer oligo-anchor R (5′-GACCACGCGTATCGATGTCGACT_16_ (A/C/G)-3′). The primers used for qRT-PCR detection are listed in Additional file 1: Table S1. The relative gene expression data were analyzed using the 2^-ΔΔCt^ method as described by Zheng et al. [42]. The results were analyzed using a one-way analysis of variance (ANOVA) statistical test. All quantitative PCR experiments were repeated in three biological replicates.

### Tissue specific expression pattern analysis of DEGs involved in reproduction

Total RNA was extracted from different tissues of the mature female adults, including the head, thorax, ovary, fat body and midgut. The tissue samples were dissected from 30 individuals. After the cDNA was obtained, qRT-PCR was performed according to the methods described above. A total of eleven genes differentially expressed during maturation and six AMPs whose transcript abundance changed after mating were used in this study.

### Change in transcriptional abundance of mating-responsive genes

To investigate the expression level of mating-responsive genes before and after mating, newly emergent individuals (1 day after emergence) of each sex were separated and the mated pairs were obtained as described above. RNA was extracted from the head or fat body of mated females 1, 12, 24 h after mating. At least 30 individuals were used for each sample and qRT-PCR experiments were repeated in three biological replicates.

## Results and discussion

### De novo sequence assembly

The cDNA samples from different stages were sequenced, and these raw data (Additional file 2: Table S2) were assembled into 39,999 unique genes (Additional file 3: Table S3). The mean contig size was 883 bp with lengths ranging from 201 to 27,791 bp (Additional file 3: Table S3 and Additional file 4: Figure S1). The contig size distribution revealed that more than half of the contigs (23,645; 59.11 %) were between 200 and 500 bp in length, whereas 34.88 % (13,951) were between 500 and 3000 bp in length (Additional file 4: Figure S1).

### Gene ontology and clusters of orthologous group classification

Gene ontology (GO) analysis was performed for functional categorization of unigenes (Additional file 5: Figure S2). Within the biological processes, the main GO terms were grouped into cellular (3362; 19 %) and metabolic (2696; 15 %; Additional file 5: Figure S2A). As for the molecular function category, binding (3663; 50 %) constituted the largest group, followed by catalytic activity (2096; 29 %; Additional file 5: Figure S2B). In the previously sequenced *B. dorsalis* transcriptome obtained from different developmental stages, the metabolic process group (35 %) was much larger than the cellular process group (16 %) [6]. The primary reason for this difference might be that the metabolic activities of insects during larval development is greater than in the adult stage. Many complicated physiological processes occurs during molting and metamorphosis, such as a molting cascade similar to the larval molt, histolysis of larval tissues, remodeling and formation of adult tissues, etc. [43, 44].

Assignments of clusters of orthologous groups (COG) were performed to further evaluate the completeness of the transcriptome and the effectiveness of our annotation process. The annotated sequences were grouped into 25 major functional classes (Additional file 6: Figure S3). Among them, the majority of the clusters were “Signal transduction mechanisms” (795; 13.01 %), “General function prediction only” (742; 12.14 %), whereas “Cell motility” (17; 0.28 %) and “Nuclear structure” (28; 0.46 %) represented the smallest groups (Additional file 6: Figure S3). Our transcriptome will aid further research focused on signal transduction in tephritid files.

### Transcriptional changes during female maturation

**Fig. 1:**
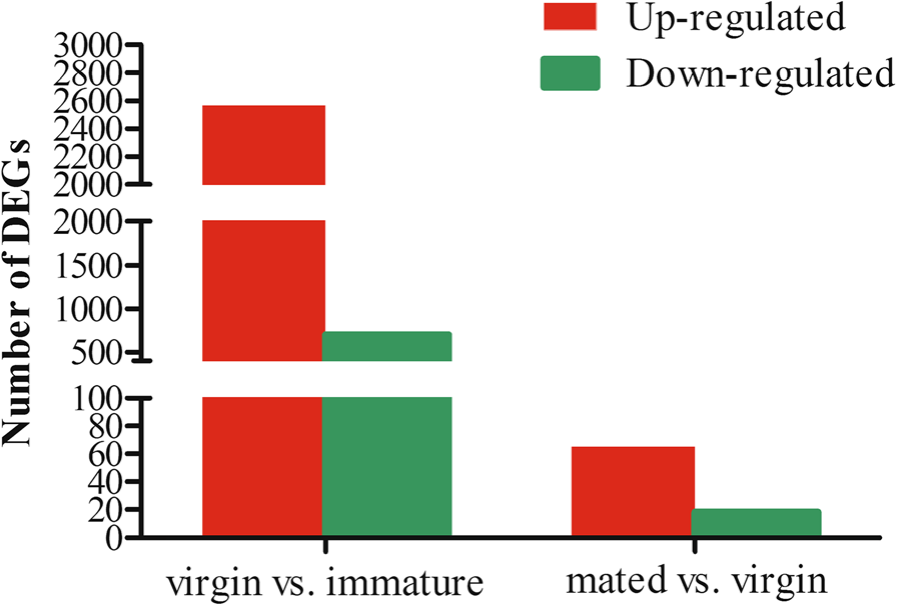
Comparisons of differentially expressed genes (DEGs) between the mature virgin and immature samples, as well as between the mated and mature virgin samples. The number of up-regulated genes is represented in the red column, while down-regulated genes are shown in green

To identify transcripts that may play a role in female maturation, differentially expressed gene sequences between the mature and immature libraries were identified with a total of 3264 DEGs (FDR < 0.001, Fig. 1 & Additional file 7: File S1). Among them, 2567 transcripts were more abundant in the mature females, while 697 were more abundant in immature females. Our results are different from those revealed by the adult transcriptome of *C. capitata* obtained with a microarray, in which only 811 transcripts displayed significant changes during female maturation. Furthermore, in the transcriptome of *C. capitata*, the transcripts with greater abundance were found in the immature females (462) instead of the mature individuals (349) [11]. These differences may be attributed to the use of different sequencing methods and the different sexual maturation time utilized by these two species. For example, *B. dorsalis* is sexually mature after 14 days, while *C. capitata* can mate after only 4 days [12, 13]. Therefore, more genes are possibly implicated in the sexual maturation in *B. dorsalis*.

**Fig. 2:**
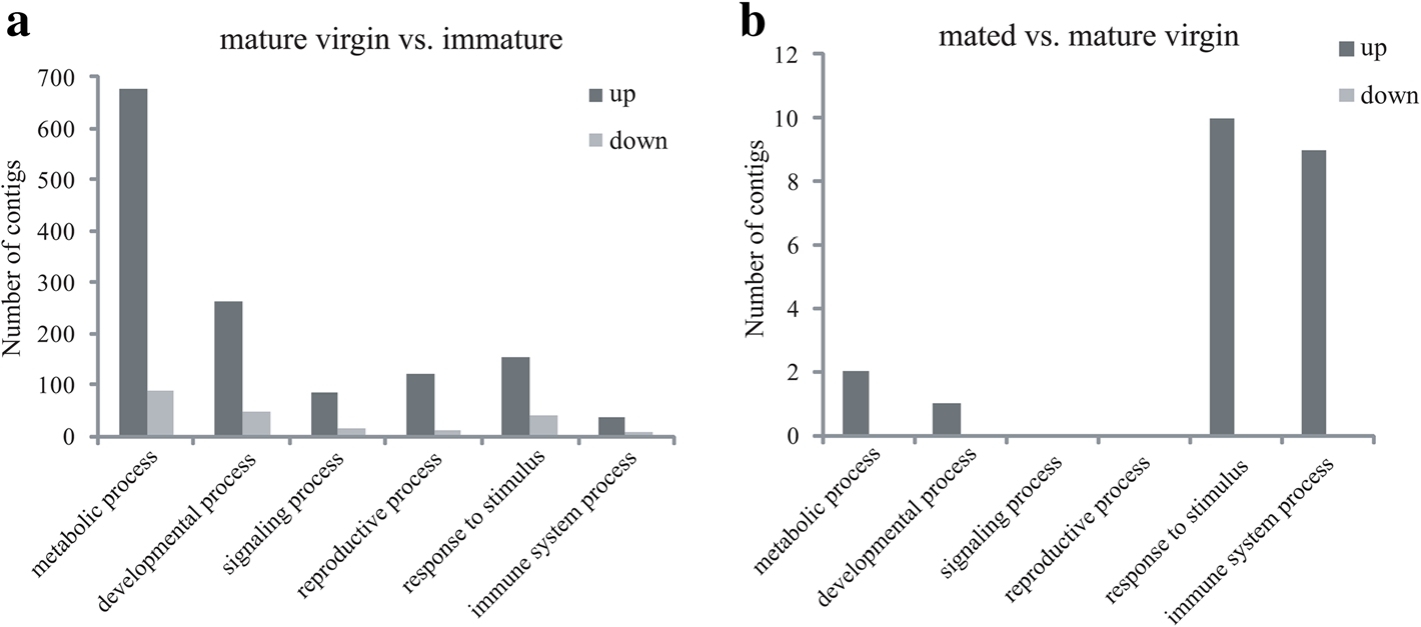
GO classification of the DEGs to demonstrate changes in transcript abundance with maturation and mating. GOEAST was used for GO term enrichment analysis, and the relative significant level of enrichment of the differently expressed gene to all genes was calculated with hypergeometric statistical test method, then the GO term whose FDR < 0.001 was considered to be significant enriched. The number of enriched transcripts for GO annotated sequences was revealed in the various samples of different biological categories. **a** Mature virgin females compared to immature females, and **b** mated females compared to mature virgin females

Analysis of enriched biological process GO terms with significant differences (*p* < 0.05) during female maturation was performed. We focused on the physiological processes that appear to play essential roles in maturation, including reproductive, signaling, developmental, metabolic, immune processes as well as response to stimulus (Fig. 2a). A table of the identified genes is provided in the supplementary data (Additional file 8: Table S4). Results revealed that differentially expressed genes in the metabolic and developmental processes are a much larger group than in other processes, which is similar to the results in *C. capitata* that indicated the most enriched terms in mature females were categorized in the metabolic process group [11]. These data indicate that a variety of metabolic activities and developmental affairs usually occur during oogenesis and ovary maturation. Additionally, enrichment analysis of the KEGG pathways for the differentially expressed genes during maturation was also performed. Results showed that the number of differentially expressed genes in the metabolism pathways are the largest groups, including Carbohydrate metabolism, Energy metabolism, Nucleotide metabolism, Lipid metabolism, Metabolism of cofactors and vitamins, Amino acid metabolism, etc. (Additional file 9: File S2). This is similar with the result of GO enrichment analysis.

### Transcriptional changes of mating-responsive genes in females

To identify transcripts that may have been involved in female mating, differentially expressed genes between the mated and mature virgin females were identified. Out of 83 transcripts that displayed significant transcriptional changes between virgin mature and mated females 24 h post-mating, 65 (78 %) were more abundant in mated females, while only 18 (22 %) were more abundant in virgin mature females (FDR < 0.001, Fig. 1 & Additional file 7: File S1). This is similar with the result found in *C. capitata* and *D. melanogaster* [11, 27, 28]. Only 32 and 28 transcripts were altered in abundance 24 h after mating in *C. capitata* and *D. melanogaster*, respectively. However, in a study of *Drosophila* using DNA microarray analysis, 539 transcripts are differentially expressed in the lower reproductive tracts [25]. One reason for the variable changes in transcription could be that like previous studies by Lawniczak and Begun [28] in *Drosophila*, our study focused on broader changes of gene expression in the whole body, while a study by Mack et al. [25] in *Drosophila* concentrated on the lower reproductive tracts, where significant changes take place following mating. Furthermore, the time point for sample collection after mating in both our study and that of the medfly was 24 h [11], that in the Lawniczak and Begun [28] analysis was 1–3 h, whereas the Mack et al. [25] examined several time points (0, 3, 6 and 24 h post-mating). It was demonstrated in previous studies that post-mating transcriptional changes are highly variable at different time points, thus possibly explaining transcriptional differences among these studies [25].

Statistically significant differences (*p* < 0.05) among enriched biological process GO terms during mating included those involved in the response to stimulus, as well as immune system, developmental, cellular, biological regulation and metabolic processes. Of these transcripts, those that are related to the response to stimulus and immune system process are the largest group (Fig. 2b). A table of these genes is provided in the supplementary data (Additional file 10: Table S5). Similar results were found in *A. mellifera* where the genes involved in stress response were significantly regulated following mating [45]. Additionally, an immune response was stimulated by mating in *Drosophila,* and it might it arise from sexually antagonistic interaction between the sexes [46, 47]. Furthermore, enrichment analysis of KEGG pathways for the mating responsive genes was also performed (Additional file 9: File S2). Our results showed that the metabolism pathways were the main groups, including carbohydrate metabolism, transport and catabolism, glycan biosynthesis and metabolism, lipid metabolism, metabolism of cofactors and vitamins. The different results between KEGG enrichment and GO enrichment might be caused by the different database used in the analysis.

#### Genes involved in oogenesis and ovary maturation

**Fig. 3:**
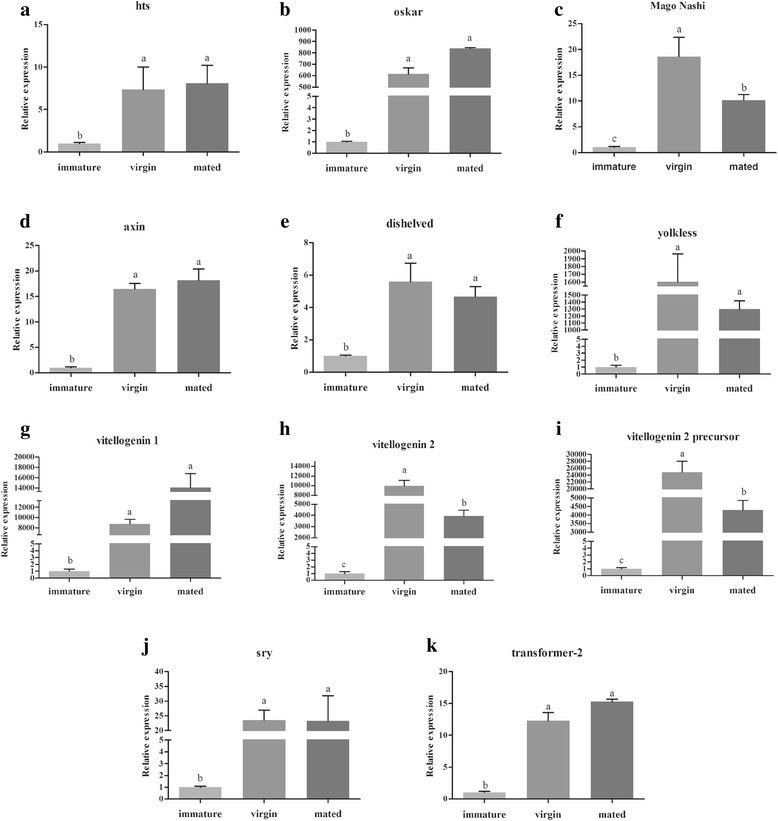
qRT-PCR confirmation of the differentially expressed genes between mature virgin females and the immature individuals. Total RNAs from *B. dorsalis* at three different physiological stages were isolated, including the immature (newly emergent within 24 h), virgin (14-day-old before mating) and mated (14-day-old post-mating). Relative transcript levels are calculated by real-time PCR using the 16 s rRNA gene as the standard control. Different letters represent significant differences (*p* < 0.05, Duncan’s test) among samples. Three independent biological replicates were performed for each sample

**Fig. 4:**
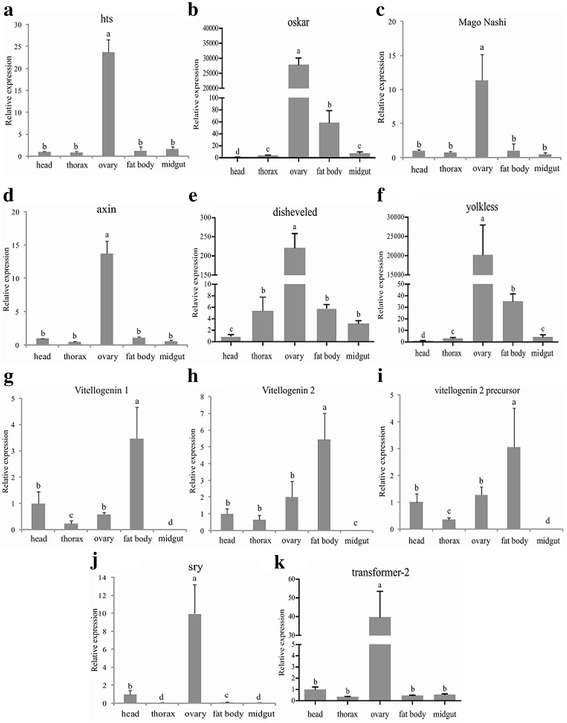
Differentially expressed genes between mature virgin females and immature individuals in various tissues. qRT-PCR was used to analyze mRNA levels of differentially expressed genes, including those found in the head, thorax, ovary, fat body and midgut. Relative transcript levels are calculated by real-time PCR using the 16 s rRNA gene as the standard control. Various letters indicate significant differences in the expression level (*p* < 0.05, Duncan’s test). Three biological replicates were performed

From DEG analysis of the samples from immature (newly eclosed) vs. mature virgin (sexually mature before mating) individuals, we identified seven genes that reportedly participate in oogenesis and ovary maturation in insects (Additional file 8: Table S4). Interestingly, two genes that were previously found to play important roles in Wnt signaling pathways and an additional two involved in sex determination were also identified in this DEG library [48–51]. The subsequent qRT-PCR validated this result (Fig. 3). All of these genes were significantly highly expressed at the sexually mature and post-mating stage, which is consistent with our transcriptome data, indicating that they are involved in physiological processes of mature females as is the case in other insects [52–54] (Fig. 3). Additionally, we analyzed expression of these genes in several tissues to elucidate their roles and found that most are highly enriched in the ovary, including *hu-li tai shao (hts)*, *oskar*, *mago nashi*, *yolkless (yl)*, *disheveled, axin*, *sry* and *transformer-2* (Fig. 4). The expression level of *oskar* in the ovary is approximately 50 fold higher than that in the fat body and 300 fold in the midgut. Notably, three *vitellogenin* genes can be detected in the head, thorax, ovary, fat body, while they are more abundantly expressed in fat body (Fig. 4). These results are explained below by analyzing their roles determined for other insects.

*hts* was reported to modulate actin polymerization in neurons, sharing a conserved function with mammalian adducing in actin-capping activity [55]. One isoform of *hts,* Ovhts-RC, was shown to be distributed along a cortical anterior-posterior gradient during *Drosophila* late-oogenesis to modulate spatially restricted actin filament growth at the oocyte cortex while alterations in Ovhts-RC led to actin overgrowth in the oocyte [52]. A previous report indicated that *oskar* participated in regulating pole plasm assembly, and a long form of *oskar* played an important role in yolk endocytosis and F-actin projection at the posterior pole [56]. Recently, it was found that *oskar* functions upstream of D-EndoB in the polarized endocytic activity of the yolk uptake process in *Drosophila* oocytes [57]. It was also found that *mago nashi* interacts with Tsunagi/Y14 and other proteins, forming a multiprotein complex to play an essential role in localization of *oskar* mRNA within the oocyte in *Drosophila* [58]. Recent analyses demonstrated that *mago nashi* forms a complex with Tsunagi/Y14 and Ranshi to influence oocyte differentiation within the posterior pole of the presumptive oocyte in *Drosophila* [53]. *Vitellogenin,* also known as *yolk protein,* has been well studied in insects, especially in *Drosophila*, *Aedes aegypti*, *Anopheles gambiae* and *C. capitata* [54, 59–62]. In these species, it is expressed in the fat body, secreted into the hemolymph and subsequently sequestered by oocytes to facilitate the transport of carbohydrates, lipids and other nutrients to the ovaries [63]. In the transcriptome we obtained, three *vitellogenin* genes were identified, including *vitellogenin 1, vitellogenin 2* and *vitellogenin 2 precursor*, all of which are up-regulated in sexually mature females, indicating that they are essential for *Bactrocera* sexual maturation. The gene *yolkless*, commonly known as the *vitellogenin receptor*, belongs to the apolipoproteins receptor (LDLR) superfamily [64]. *Drosophila yolkless* is expressed very early during the development of the oocyte at both transcriptional and translational level, long before vitellogenesis begins. Previous studies found that *yolkless* mutants failed to incorporate yolk proteins during oogenesis [65]. Our findings are validated in the qRT-PCR results, revealing that these genes were enriched in the ovary or fat body.

Furthermore, we found *disheveled* and *axin* (isoform A) in our transcriptome, both of which are core components of Wnt signaling pathways (Additional file 8: Table S4). They are also required for cell movement and FAK regulation during ovarian morphogenesis, which may explain why both genes are abundantly expressed in mature *B. dorsalis* females [48] (Fig. 3).

Notably, two genes involved in sex determination were also identified, including sex-determining region y protein (*sry*) and transformer-2 (*tra-2*). It has been reported that *sry* plays a role in mammalian sex determination, reduced or delayed *sry* expression impairs testis development. This indicates a potential role of *sry* dysregulation in human intersex disorders [49]. Additionally, *tra-2* was also reported to be an essential switch for the sex determination cascade in dipteran insect species [50, 51]. Knockdown of *tra-2* leads to the production of male-only progeny in *B. dorsalis* [66]. In the present study, we identified these two genes and found they were up-regulated in sexually mature females compared to their newly eclosed counterparts, which is the first evidence that sex determination related genes may also function during sex maturation.

#### Genes involved in the post-mating response of females

**Fig. 5:**
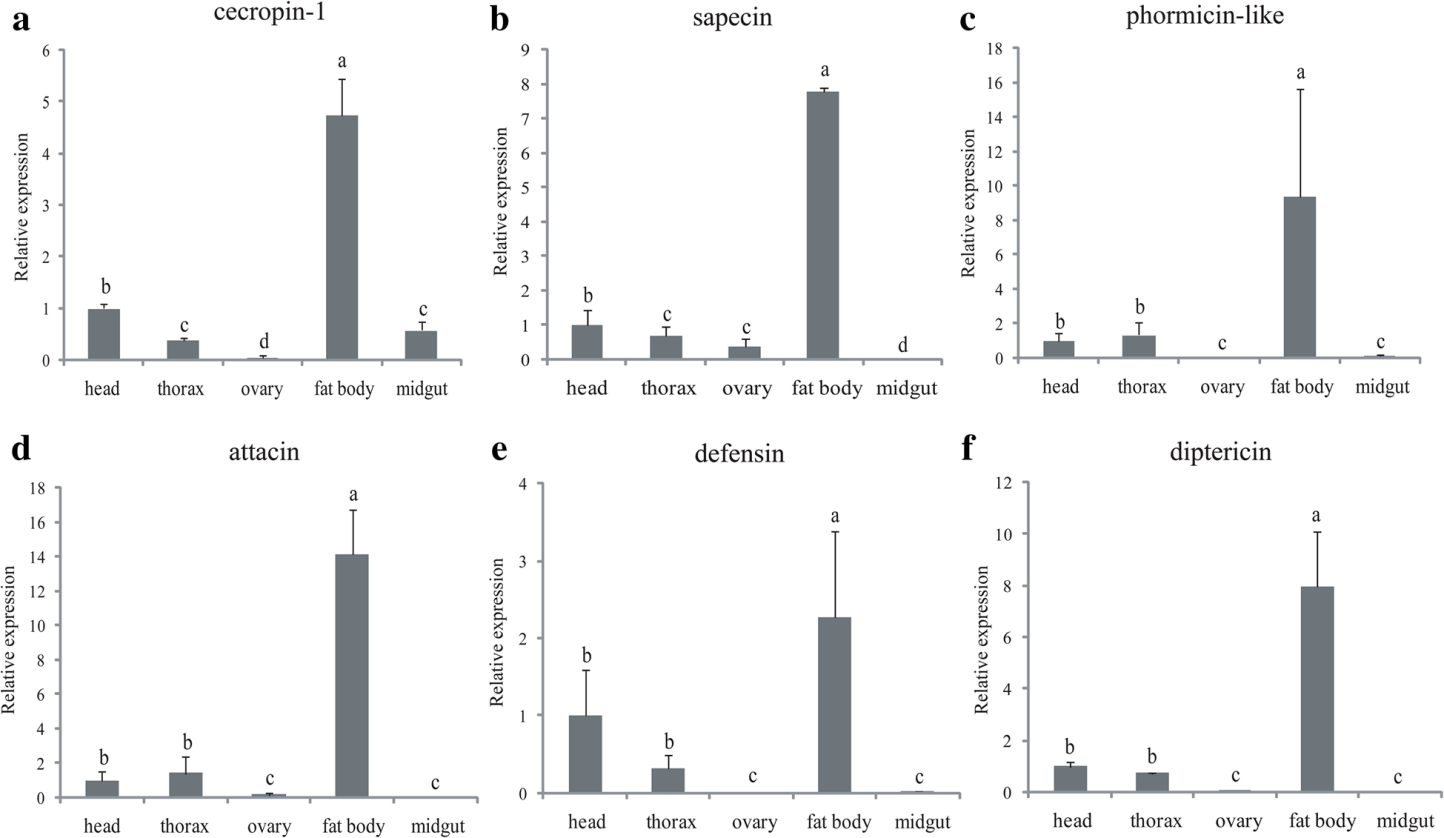
Expression pattern of the immune-related genes between mated females and the mature virgin individuals in different tissues, including the head, thorax, ovary, fat body and midgut. Relative transcript levels of six *antimicrobial peptide* are calculated by real-time PCR using the 16 s rRNA gene as the standard control. Different letters indicate significant differences (*p* < 0.05, Duncan’s test) among samples and three independent biological replicates were performed for each sample

Upon mating, significant expression changes occurred in genes involved in the immune or stress response (Fig. 2b). The immune response stimulated by mating might arise from sexually antagonistic interaction between the sexes [27, 28, 46, 47]. Several up-regulated genes from the DEG analysis showed significant increases in transcript abundance in females, including two PRRs (peptidoglycan-recognition protein SB1-like and peptidoglycan-recognition protein LB-like) and six antimicrobial peptides (AMPs) (*defensin, diptericin, phormicin-like, sapecin, cecropin-1* and *attacin-C-like*) (Additional file 10: Table S5). To investigate the expression pattern of these genes, the expression levels of AMPs in different tissues were analyzed by qRT-PCR. The results showed that all were highly enriched in the fat body, which is usually an important organ involved in the immune response in insects (Fig. 5). The expression pattern of these genes indicates that they may participate in the immune response after mating in *B. dorsalis*.

**Fig. 6:**
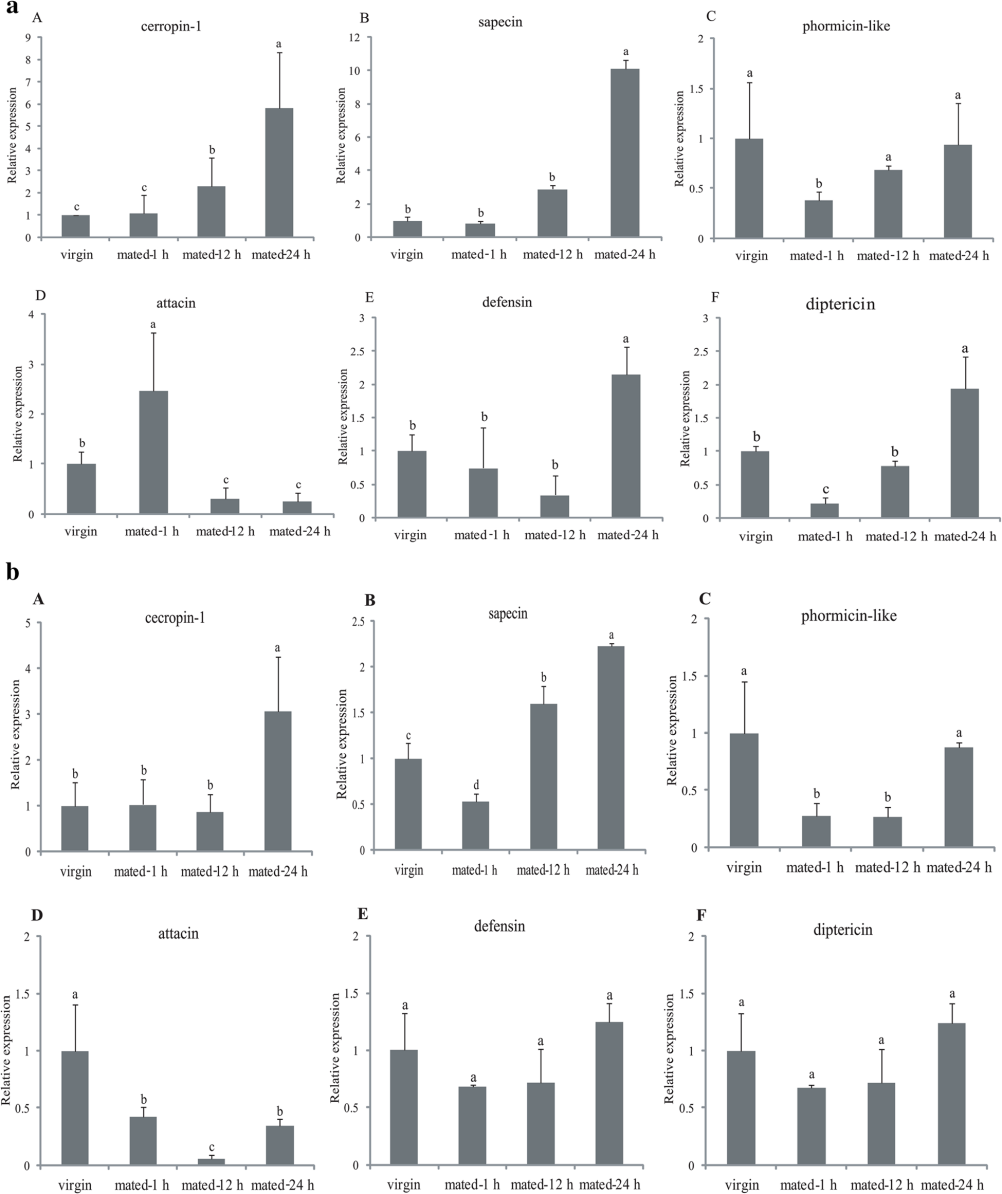
The effect of mating on the expression level of immune-related genes in the fat body (**a**) and head (**b**) of *B. dorsalis* females. RNA was extracted from the head or fat body of mature vigin females as well as mated females at 1, 12, 24 h after mating. Relative transcript levels of six *antimicrobial peptide* are calculated by real-time PCR using the 16 s rRNA gene as the standard control. Various letters represent significant differences of the expression level of genes (*p* < 0.05, Duncan’s test). Three biological replicates were performed

To further validate, the changes in expression at different time points after mating were analyzed in both the fat body and head. All genes except *attacin-C-like* and *phormicin-like* were significantly up-regulated 24 h after mating in the fat body approximately 2–10 fold (Fig. 6a). This is partly supported by the previous studies in *D. melanogaster,* which showed that the immune response is activated in mated females by mating and associated sex peptides transfer [28, 46, 47]. Upon mating, potent signal molecules from the males enter the females and the behavior and physiology of the mated females shifted from reproductive receptivity to fecundity. The immune response was induced in the females and it might arise from sexually antagonistic interaction between the sexes [45, 47]. However, this is different from the results of the previous report on *C. capitata*, which revealed no significant changes of transcript abundance were detected in immune-related genes after mating, except for a large reduction in *defensin* in the female abdomen [11]. This data suggests that post mating changes differ among species [33]. Additionally, the expression level of most AMPs did not significantly change during the time course in the female head 24 h post-mating, except for *sapecin* and *cecropin-1,* both of which significantly increased in transcript abundance, by 2.3 and 3.0 fold, respectively (Fig. 6b). It is clear that the fat body is the main tissue where the immune response takes place and not the head.

## Conclusions

This study significantly advances the characterization of the sexual maturation and mating process at the molecular level in the oriental fruit fly, a major invasive agricultural tephritid pest. Numerous transcripts displayed significant changes in abundance during maturation of the female oriental fruit fly and generally mirrored the physiological and behavioral alterations in female flies. Multiple reproductive genes were differentially expressed between mature virgin and immature individuals, notably including two genes involved in sex determination, possibly indicating they have important roles during sexual maturation. However, modest post-mating changes were also observed in females. The difference in transcriptional changes pertaining to sexual maturation and mating, compared to *C. capitata*, is perhaps a result of different reproductive strategies employed by these species.

Our data reveals the existence of additional potential molecular targets for *B. dorsalis* control, and may aid in future studies of reproductive mechanisms in fruit flies. Furthermore, our findings provide a comprehensive resource for exploring the complex molecular mechanisms driving the evolution of reproductive processes, and will likely facilitate the development of effective and eco-friendly pest control strategies.

## Availability of supporting data

This Transcriptome Shotgun Assembly project has been deposited at DDBJ/EMBL/GenBank under the accession GEEA00000000. Other data sets supporting the results of this article are included in the additional files.

## Additional files

Additional file 1: Table S1. Primers used for qRT-PCR. (DOC 36 kb) Additional file 2: Table S2. Summary of the Solexa sequencing statistics of *B. dorsalis* adult transcriptome. (DOCX 14 kb) Additional file 3: Table S3. Summary of the *B. dorsalis* adult transcriptome. (DOCX 12 kb) Additional file 4: Figure S1. Assembled contig length distribution of the transcriptome. The x-axis shows contig size and the y-axis indicates the number of contigs for each given size. (TIF 1138 kb) Additional file 5: Figure S2. Gene ontology (GO) analyses of transcriptome sequences predicted to participate in: (A) biological processes and (B) molecular functions. Data are presented as level 3 GO categorization for molecular function and level 2 GO categorization for biological process. Classified gene objects are showed as percentages (in brackets) of the total number according to the GO assignments. (ZIP 1448 kb) Additional file 6: Figure S3. Histogram presentation of Clusters of orthologous groups (COG) classification. A total of 6111 predicted unigenes were classified in 25 COG categories. (TIF 2126 kb) Additional file 7: File S1. Genes differentially expressed between mature virgin females and immature individuals, and between mated females and mature virgins. Reads, RPKM and the fold values for each comparison group (mature virgin vs. immature, and mated vs. mature virgin) is shown. (XLS 674 kb) Additional file 8: Table S4. Oriental fruit fly assembled sequences of DEGs (mature virgin vs. immature) with best-hit matches to dipteran genes involved in the sexual maturation. (DOC 56 kb) Additional file 9: File S2. Enrichment analysis of KEGG pathways for the differentially expressed genes. (XLS 25 kb) Additional file 10: Table S5. Oriental fruit fly assembled sequences of DEGs (mated vs. mature virgin) with best-hit matches to dipteran genes involved in the immune system process or response to stimulus. (DOC 57 kb)
