# Supplementary material for: RNA sequencing to characterize transcriptional changes of sexual maturation and mating in the female oriental fruit fly Bactrocera dorsalis
Source: BMC Genomics. 2016 Mar 5;17:194. doi: 10.1186/s12864-016-2532-6 (PMC4779581; doi:10.1186/s12864-016-2532-6)
Supplement: Additional file 1: Table S1. — Primers used for qRT-PCR. (DOC 36 kb) [file 12864_2016_2532_MOESM1_ESM.doc]

**Table S1** Primers used for qRT-PCR.

| Primer name | (5′→3′) nucleotide sequence |
| --- | --- |
| axin RTF  axin RTR  dishevelled RTF  dishevelled RTR  hltsRTF  hltsRTR  oskar RTF  oskar RTR  Mago Nashi RTF  Mago Nashi RTR  sryRTF  sryRTR  transformer-2RTF  transformer-2RTR  yolklessRTF  yolklessRTR  vitellogenin 1RTF  vitellogenin 1RTR  vitellogenin 2RTF  vitellogenin 2RTR  vitellogenin 2 precursor RTF  vitellogenin 2 precursor RTR  phormicin-like RTF | TGAACGATTTGGGATTGG  CGCTACCGAAGAGCAAGG  CTGCTCGGAGTTGCCAGTAA  AACGCCAGTATTCGCTAAAC  CCACCTGAGGATTTGGAGA  CAACATACGCATGAGTGCC  GAAGCAGCCTAATCCATACA  TCATAGTCGGAATCTGTCGT  ACTGCGCTACGCCAACAA  ATCGCCTATGACAATTTCCAAC  GTGTCGGGTGGACAAACCT  ACTATGTAGCGGGCTGGGT  GTCGCAGTTACACCAAGTCG  TGCCGTGAGGCTGATCTACT  ACCACAGATACCGAAACGA  CTAAAGCCAATGAGTAGGGAC  CAGCGAAAGATTGAGAAGC  GTTTGGCAGTTTGGACGAG  TGAGGAGGCTGCCGAGTT  GACCGAAATGAGGTTGTTGTTG  CGAAGAGGACTACAGCGAATC  CGAGCAAAGCATAGCGTTTA  TGGAGCAGATGTTGGAGAA |
| phormicin-like RTR | GGTTGCCACGAAGTAGACA |
| sapecinRTF  sapecinRTR | AAGAGCCTGCGGTGGAGT  CACGGTTGCCACGGAGTA |
| cecropin-1RTF  cecropin-1RTR  attacin-C-like RTF  attacin-C-like RTR  diptericin RTF  diptericin RTR  defensinRTF  defensinRTR | CATCTTCTTGGCTGTGGTG  CTTGAACGGCGGCATCTC  GCTCTTCTTGCTCGCCTTAG  AGCCACCAACACCAACTTCA  CCCCAAAGACAGCCTCAG  CGCCCAAATGTTGCGAAT  GCCGTGGAAGCAGAGGAT  AAGCAGCAAGCAATGGAATAT |
| 16srRNARTF | CTCGTCCAACCGTTCATACC |
| 16srRNARTR | CTGACCTGCCCACTGAAGTT |
|  |  |
