# Supplementary material for: RNA sequencing to characterize transcriptional changes of sexual maturation and mating in the female oriental fruit fly Bactrocera dorsalis
Source: BMC Genomics. 2016 Mar 5;17:194. doi: 10.1186/s12864-016-2532-6 (PMC4779581; doi:10.1186/s12864-016-2532-6)
Supplement: Additional file 2: Table S2. — Summary of the Solexa sequencing statistics of B. dorsalis adult transcriptome. (DOCX 14 kb) [file 12864_2016_2532_MOESM2_ESM.docx]

Table S2 Summary of the Solexa sequencing statistics of *B. dorsalis* adult transcriptome.

| **Samples** | **1-1** | **1-2** | **2-1** | **2-2** | **3-1** | **3-2** |
| --- | --- | --- | --- | --- | --- | --- |
| Raw Reads (pair) | 6498198 | 6057481 | 6172207 | 7325905 | 5209227 | 6312789 |
| Raw data | 1299639600 | 1211496200 | 1234441400 | 1465181000 | 1041845400 | 1262557800 |
| Clean reads(pair) | 6467645 | 5973784 | 6085817 | 7210233 | 5092640 | 6214727 |
| Clean data | 1293529000 | 1194756800 | 1217163400 | 1442046600 | 1018528000 | 1242945400 |

1-1,1-2: Two replicates of samples for immature females;

2-1,2-2: Two replicates of samples for mature virgin females;

3-1,3-2: Two replicates of samples for mated females.
