# Supplementary material for: RNA sequencing to characterize transcriptional changes of sexual maturation and mating in the female oriental fruit fly Bactrocera dorsalis
Source: BMC Genomics. 2016 Mar 5;17:194. doi: 10.1186/s12864-016-2532-6 (PMC4779581; doi:10.1186/s12864-016-2532-6)
Supplement: Additional file 3: Table S3. — Summary of the B. dorsalis adult transcriptome. (DOCX 12 kb) [file 12864_2016_2532_MOESM3_ESM.docx]

Table S3 Summary of the *B. dorsalis* adult transcriptome.

| Total base pairs (bp) | 350,865,036 |
| --- | --- |
| Average read length (bp) | 200 |
| Number of contigs | 39999 |
| Average contig length (bp) | 883 |
| Range of contig length (bp) | 201 – 27791 |
| N50 length (bp) | 1839 |
