# Supplementary figures and images for: RNA sequencing to characterize transcriptional changes of sexual maturation and mating in the female oriental fruit fly Bactrocera dorsalis
Source: BMC Genomics. 2016 Mar 5;17:194. doi: 10.1186/s12864-016-2532-6 (PMC4779581; doi:10.1186/s12864-016-2532-6)

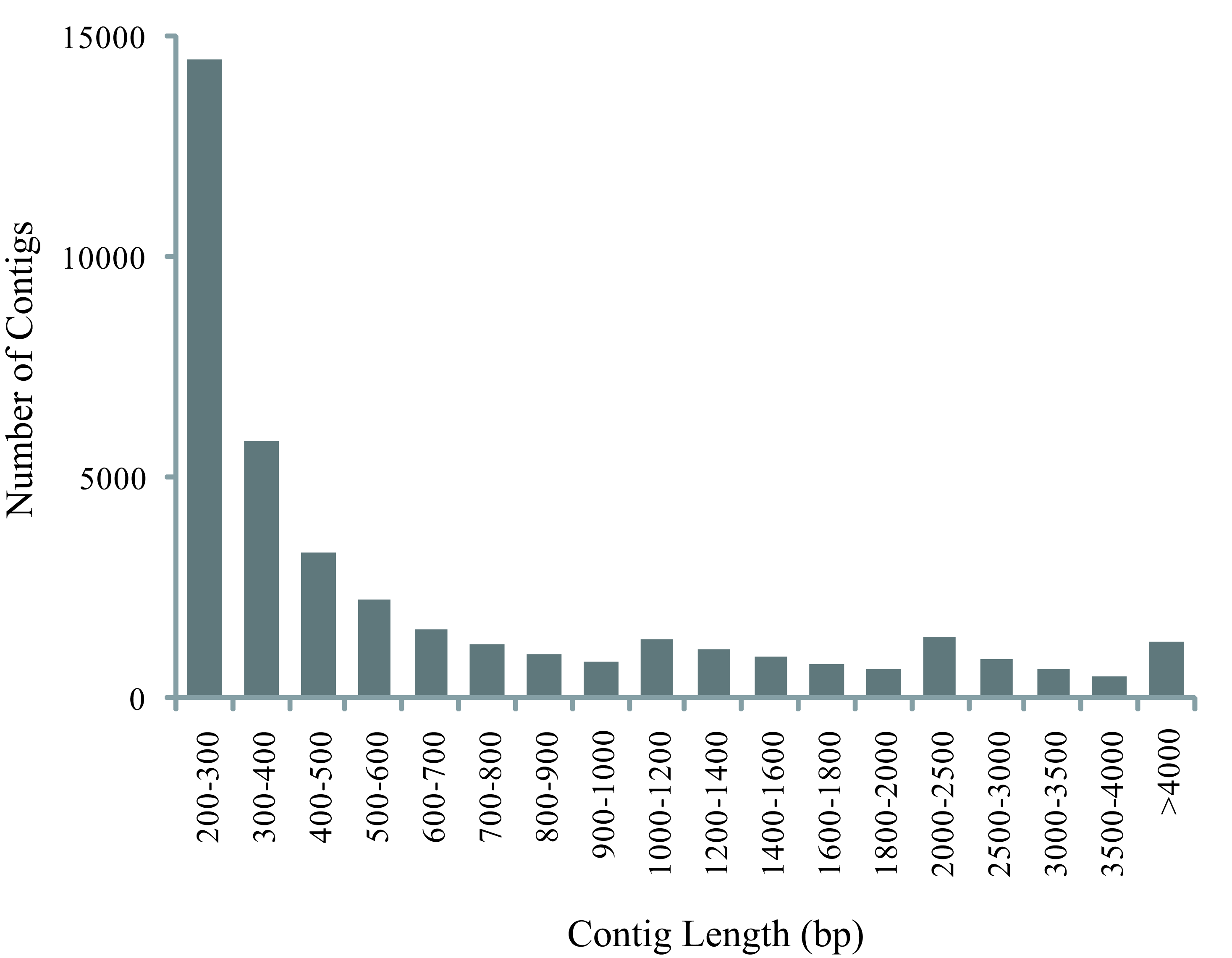

Supplement: Additional file 4: Figure S1. — Assembled contig length distribution of the transcriptome. The x-axis shows contig size and the y-axis indicates the number of contigs for each given size. (TIF 1138 kb) [file 12864_2016_2532_MOESM4_ESM.tif]

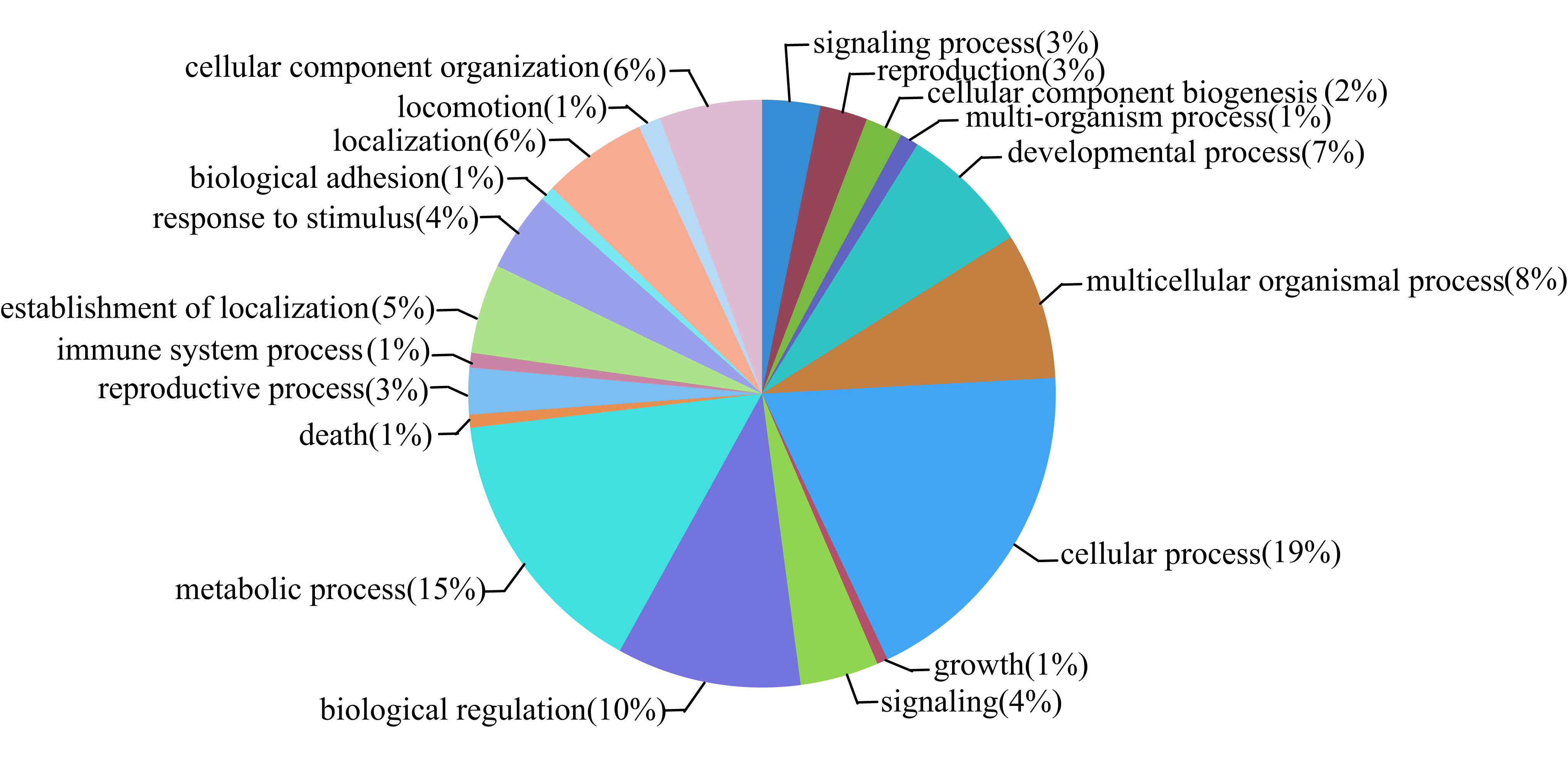

Supplement: Additional file 5: Figure S2. — Gene ontology (GO) analyses of transcriptome sequences predicted to participate in: (A) biological processes and (B) molecular functions. Data are presented as level 3 GO categorization for molecular function and level 2 GO categorization for biological process. Classified gene objects are showed as percentages (in brackets) of the total number according to the GO assignments. (ZIP 1448 kb) [file 12864_2016_2532_MOESM5_ESM.zip › Fig S2A.tif]

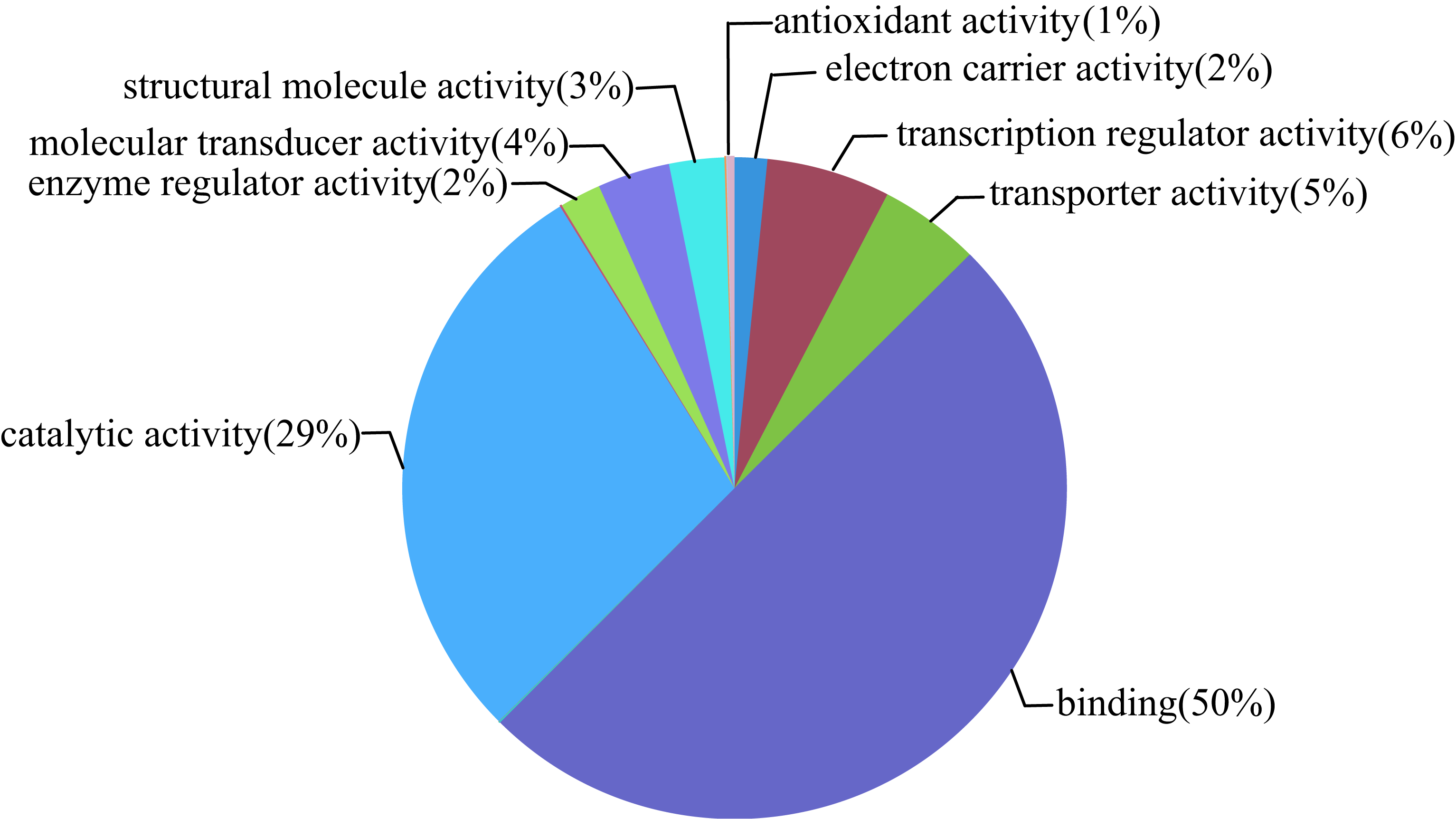

Supplement: Additional file 5: Figure S2. — Gene ontology (GO) analyses of transcriptome sequences predicted to participate in: (A) biological processes and (B) molecular functions. Data are presented as level 3 GO categorization for molecular function and level 2 GO categorization for biological process. Classified gene objects are showed as percentages (in brackets) of the total number according to the GO assignments. (ZIP 1448 kb) [file 12864_2016_2532_MOESM5_ESM.zip › Fig S2B.tif]

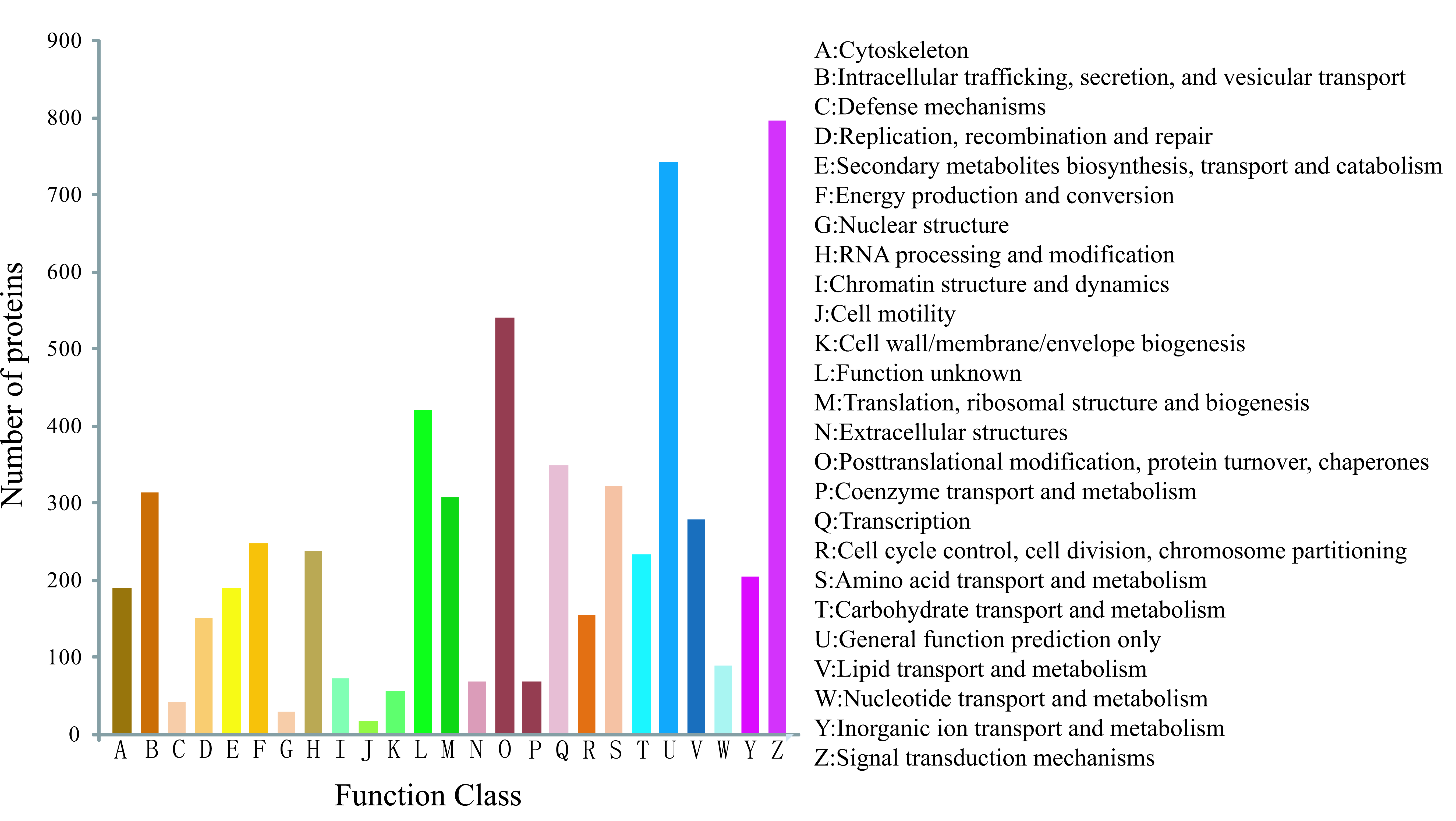

Supplement: Additional file 6: Figure S3. — Histogram presentation of Clusters of orthologous groups (COG) classification. A total of 6111 predicted unigenes were classified in 25 COG categories. (TIF 2126 kb) [file 12864_2016_2532_MOESM6_ESM.tif]
