# Supplementary material for: RNA sequencing to characterize transcriptional changes of sexual maturation and mating in the female oriental fruit fly Bactrocera dorsalis
Source: BMC Genomics. 2016 Mar 5;17:194. doi: 10.1186/s12864-016-2532-6 (PMC4779581; doi:10.1186/s12864-016-2532-6)
Supplement: Additional file 8: Table S4. — Oriental fruit fly assembled sequences of DEGs (mature virgin vs. immature) with best-hit matches to dipteran genes involved in the sexual maturation. (DOC 56 kb) [file 12864_2016_2532_MOESM8_ESM.doc]

**Table S4.** Oriental fruit fly assembled sequences of DEGs (mature virgin vs. immature) with best-hit matches to Dipteran genes involved in reproductive process.

| **Gene name** | **Gene ID** | **Length**  **(bp)** | **Species** | **Subject ID** | **E-value** | **Identity (%)** |
| --- | --- | --- | --- | --- | --- | --- |
| hu li tai shao | comp63038_c0_seq1 | 2235 | *C.capitata* | XP_004517361.1 | 0 | 97 |
|  |  |  | *D. melanogaster* | NP_725886.2 | 0 | 83 |
| yolkless | comp66315_c0_seq2 | 5793 | *C.capitata* | XP_004529642.1 | 0 | 83 |
|  |  |  | *D. melanogaster* | NP_996433.1 | 0 | 55 |
| oskar | comp53308_c0_seq1 | 1575 | *C.capitata* | XP_004529162.1 | 0 | 67 |
|  |  |  | *D. melanogaster* | [NP_996186.1](http://www.ncbi.nlm.nih.gov/protein/45553317?report=genbank&log$=prottop&blast_rank=19&RID=W949HNBG01R) | 7e-68 | 61 |
| Mago nashi | comp52652_c0_seq2 | 486 | *C.capitata* | XP_004517546.1 | 1e-103 | 99 |
|  |  |  | *D. melanogaster* | NP_476636.1 | 2e-101 | 97 |
| vitellogenin-1 | comp59108_c0_seq1 | 1383 | *C.capitata* | XP_004524985.1 | 0 | 83 |
|  |  |  | *D. melanogaster* | [NP_511103.1](http://www.ncbi.nlm.nih.gov/protein/17530879?report=genbank&log$=prottop&blast_rank=19&RID=W94UZ4ME01R) | 7e-157 | 58 |
| vitellogenin-2 | comp62442_c0_seq1 | 1278 | *C.capitata* | XP_004524984.1 | 0 | 83 |
|  |  |  | *D. melanogaster* | [NP_511102.3](http://www.ncbi.nlm.nih.gov/protein/161077703?report=genbank&log$=prottop&blast_rank=23&RID=W94VKHA001R) | 2e-145 | 52 |
| vitellogenin-2  precursor | comp62911_c0_seq1 | 1314 | *C.capitata* | XP_004530498.1 | 0 | 75 |
|  |  |  | *D. melanogaster* | [NP_511103.1](http://www.ncbi.nlm.nih.gov/protein/17530879?report=genbank&log$=prottop&blast_rank=20&RID=W94XPJ7S01R) | 2e-158 | 57 |
| sry* | comp65211_c0_seq2 | 1236 | *C.capitata* | XP_004537902.1 | 2e-165 | 90 |
|  |  |  | *D. melanogaster* | [NP_524735.1](http://www.ncbi.nlm.nih.gov/protein/17975569?report=genbank&log$=prottop&blast_rank=12&RID=W945G5BZ015) | 8e-86 | 57 |
| transformer-2* | comp55532_c0_seq1 | 814 | *C.capitata* | [NP_001266337.1](http://www.ncbi.nlm.nih.gov/protein/525343362?report=genbank&log$=prottop&blast_rank=23&RID=W93SBWS8015) | 5e-60 | 97 |
|  |  |  | *D. melanogaster* | [NP_476766.1](http://www.ncbi.nlm.nih.gov/protein/17136550?report=genbank&log$=prottop&blast_rank=93&RID=W93SBWS8015) | 7e-40 | 69 |
| disheveled** | comp61712_c0_seq1 | 1780 | *C.capitata* | XP_004527373.1 | 0 | 96 |
|  |  |  | *D. melanogaster* | NP_511118.2 | 0 | 79 |
| axin** | comp58823_c0_seq1 | 2217 | *C.capitata* | XP_004529158.1 | 0 | 93 |
|  |  |  | *D. melanogaster* | NP_733336.1 | 0 | 76 |

*C. capitata: Ceratitis capitata*; *D .melanogaster: Drosophila melanogaster*; sry: sex-determining region y protein; *: genes are involved in sex determination; **: genes are involved in Wnt pathways; the other genes are involved in oogenesis.
