# Supplementary material for: RNA sequencing to characterize transcriptional changes of sexual maturation and mating in the female oriental fruit fly Bactrocera dorsalis
Source: BMC Genomics. 2016 Mar 5;17:194. doi: 10.1186/s12864-016-2532-6 (PMC4779581; doi:10.1186/s12864-016-2532-6)
Supplement: Additional file 10: Table S5. — Oriental fruit fly assembled sequences of DEGs (mated vs. mature virgin) with best-hit matches to dipteran genes involved in the immune system process or response to stimulus. (DOC 57 kb) [file 12864_2016_2532_MOESM10_ESM.doc]

**Table S5.** Oriental fruit fly assembled sequences of DEGs ( mated vs. mature virgin) with best-hit matches to Dipteran genes involved in immune system process or response to stimulus.

| **Gene name** | **Gene ID** | **Length**  **(bp)** | **Species** | **Subject ID** | **E-value** | **Identity (%)** |
| --- | --- | --- | --- | --- | --- | --- |
| phomicin | comp55812_c0_seq1 | 378 | *C.capitata* | [XP_004537627.1](http://www.ncbi.nlm.nih.gov/protein/499013805?report=genbank&log$=protalign&blast_rank=3&RID=W3P659EN014) | 1e-23 | 67 |
|  |  |  | *D. melanogaster* | AAO72491.1 | 2e-06 | 43 |
| sapecin | comp59000_c1_seq2 | 342 | *C.capitata* | XP_004537435.1 | 3e-23 | 88 |
|  |  |  | *D. melanogaster* | AAO72492.1 | 9e-15 | 63 |
| cecropin-1 | comp55400_c0_seq1 | 213 | *C.capitata* | XP_004534334.1 | 3e-18 | 88 |
|  |  |  | *D. melanogaster* | AAB82493.1 | 4e-15 | 71 |
| attacin | comp62552_c0_seq2 | 726 | *C.capitata* | XP_004517762.1 | 1e-135 | 89 |
|  |  |  | *D. melanogaster* | NP_523745.1 | 8e-86 | 68 |
| diptericin | comp58240_c0_seq2 | 375 | *C.capitata* | XP_004536458.1 | 2e-29 | 55 |
|  |  |  | *D. melanogaster* | AF334182_1 | 1e-22 | 75 |
| defensin | comp27338_c0_seq1 | 291 | *C.capitata* | XP_012162175.1 | 1e-29 | 69 |
|  |  |  | *D. melanogaster* | AAO72500.1 | 3e-04 | 49 |
| PGRP-SB1 | comp61760_c0_seq2 | 598 | *C.capitata* | XP_004537949.1 | 1e-122 | 89 |
|  |  |  | *D. melanogaster* | CAD89131.1 | 4e-97 | 76 |
| PGRP-LB | comp58205_c0_seq2 | 816 | *C.capitata* | XP_004518326.1 | 6e-87 | 77 |
|  |  |  | *D. melanogaster* | NP_001247052.1 | 2e-38 | 49 |
| GNBP3 | comp63788_c0_seq3 | 1611 | *C.capitata* | XP_004518427.1 | 0 | 62 |
|  |  |  | *D. melanogaster* | NP_523986.2 | 2e-74 | 39 |
| IMIP* | comp59160_c0_seq1 | 402 | *C.capitata* | XP_012154590.1 | 6e-37 | 67 |
|  |  |  | *D. melanogaster* | NP_001036256.2 | 1e-22 | 56 |

*C. capitata: Ceratitis capitata*; *D .melanogaster: Drosophila melanogaster*

PGRP-SB1: peptidoglycan-recognition protein SB1;

PGRP-LB: peptidoglycan-recognition protein LB ;

GNBP3: gram-negative bacteria-binding protein 3 ;

IMIP: inducible metalloproteinase inhibitor protein. *: IMIP only belongs to the gene ontology termed “response to stimulus”, while others belong to both “response to stimulus” and “immune system process” term.
